# Programmable Spectral Filter in C-Band Based on Digital Micromirror Device

**DOI:** 10.3390/mi10030163

**Published:** 2019-02-27

**Authors:** Yunshu Gao, Xiao Chen, Genxiang Chen, Zhongwei Tan, Qiao Chen, Dezheng Dai, Qian Zhang, Chao Yu

**Affiliations:** 1 College of Science, Minzu University of China, Beijing 100081, China; gaoyunshu@126.com (Y.G.); chenqiao0117@163.com (Q.C.); daidezheng@126.com (D.D.); qianzhang1521@163.com (Q.Z.); 2 School of Electronic and Information Engineering, Beijing Jiaotong University, Beijing 100044, China; zhwtan@bjtu.edu.cn; 3 School of Electronic Engineering, Beijing University of Posts and Telecommunications, Beijing 100876, China; yu_chao@bupt.edu.cn

**Keywords:** programmable spectral filter, digital micromirror device, optical switch

## Abstract

Optical filters have been adopted in many applications such as reconfigurable telecommunication switches, tunable lasers and spectral imaging. However, most of commercialized filters based on a micro-electrical-mechanical system (MEMS) only provide a minimum bandwidth of 25 GHz in telecom so far. In this work, the programmable filter based on a digital micromirror device (DMD) experimentally demonstrated a minimum bandwidth of 12.5 GHz in C-band that matched the grid width of the International Telecommunication Union (ITU) G.694.1 standard. It was capable of filtering multiple wavebands simultaneously and flexibly by remotely uploading binary holograms onto the DMD. The number of channels and the center wavelength could be adjusted independently, as well as the channel bandwidth and the output power. The center wavelength tuning resolution of this filter achieved 0.033 nm and the insertion loss was about 10 dB across the entire C-band. Since the DMD had a high power handling capability (25 KW/cm^2^) of around 200 times that of the liquid crystal on silicon (LCoS) chip, the DMD-based filters are expected to be applied in high power situations.

## 1. Introduction

To maximize the use of fiber spectral capacity and improve network efficiency, the International Telecommunication Union (ITU) G.694.1 standard has replaced the earlier G.692 specification to eliminate inefficient optical guard bands. The next generation optical network is a flexible elastic grid network that can dynamically allocate an amount of spectral resources as needed in 12.5 GHz increments for individual channels to support different symbol rates. It appears that traditional reconfigurable optical add/drop multiplexers (ROADMs) that provide optical switching of the fixed wavelength need to be upgraded to support flexible grid channels [1]. The flexible-grid ROADMs [2] must consist of programmable channel switching devices, such as wavelength selective switches (WSSs) or flexible-grid filters.

Currently there are two main competing technologies used in commercial WSSs and filters: The micro-electrical-mechanical system (MEMS) and the liquid crystal on silicon (LCoS) spatial light modulator (SLM). With the digital micromirror device (DMD) as an electrical input, optical output MEMS performs efficient and reliable spatial light modulation. The DMD developed by Texas Instruments is an array of thousands to millions of tiny highly reflective aluminum micromirrors which can be addressed independently. It has the advantage of low cost, high power handling and a fast frame rate, so that it has become an attractive solution for modulating spectrum resources in reconfigurable telecommunication switches [3,4], dynamic spatial and image filters [5,6], tunable lasers [7,8,9,10] and equalizers for erbium-doped fiber amplifiers (EDFAs) [11]. However, DMD and MEMS based reconfigurable optical filters [12,13,14] up to date have the minimum spectral bandwidth of 25 GHz in C-band (The TrueFlex Twin Multi-Cast Switch produced by Lumentum), so that they cannot adapt to the development of the new ITU-T standard grid frequencies. Although it was reported LCoS-based WSSs [15,16] have achieved 12.5 GHz or even smaller minimum bandwidth and higher tuning resolution, the LCoS processor under high power operation starts to deteriorate in function, even acquiring permanent, irreparable damage [17]. In comparison, the DMD has a high power handling capability and the only limit of the device under high power illumination is that the aluminum micromirrors must operate below 150 °C [18]. Therefore, it is expected to greatly broaden the application of DMD based optical filters in high power situations.

In this paper, we employ a DMD combining with a high-line-density transmission grating into a 2-*f* optical system to demonstrate the programmable spectral filter with flexible center wavelength, elastic bandwidth and high power handling. The minimum bandwidth achieved was 12.5 GHz. This optical filter can become a pre-filter to obtain target spectrum or an equalizer for EDFAs. It is important to compensate lower power handling of LCoS-based switches in an optical network.

## 2. System Design

**Figure 1:**
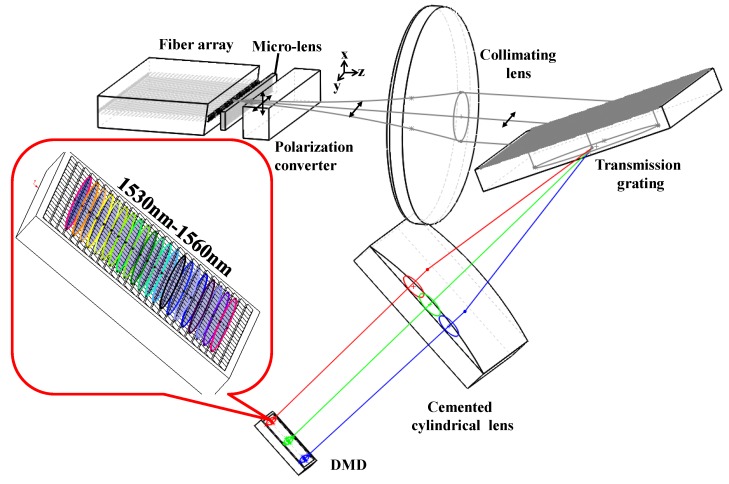
Diagram of programmable optical filter based on the digital micromirror device (DMD) chip.

Figure 1 illustrates the layout of the DMD-based optical filter which consists of a fiber-coupling microlens array, a polarization converter, two lenses, a transmission grating and a DMD. Two ports from the fiber-coupling microlens array with a 127 μm-pitch were used as an input and an output. For the high-line-density transmission grating with 1201.2 line/mm is S-polarization dependent, a polarization converter was inserted after the micro-lens to modulate the polarization state of an input beam. The DMD adopted in system consisted of 1024 × 768 mirrors on a pitch of δ = 13.68 μm with ±12° micromirror tilt by software control. It had a highly efficient steering of NIR light and an anti-reflection coated substrate which assured a front cover reflection of less than 0.5% between 1400 and 1700 nm.

**Figure 2:**
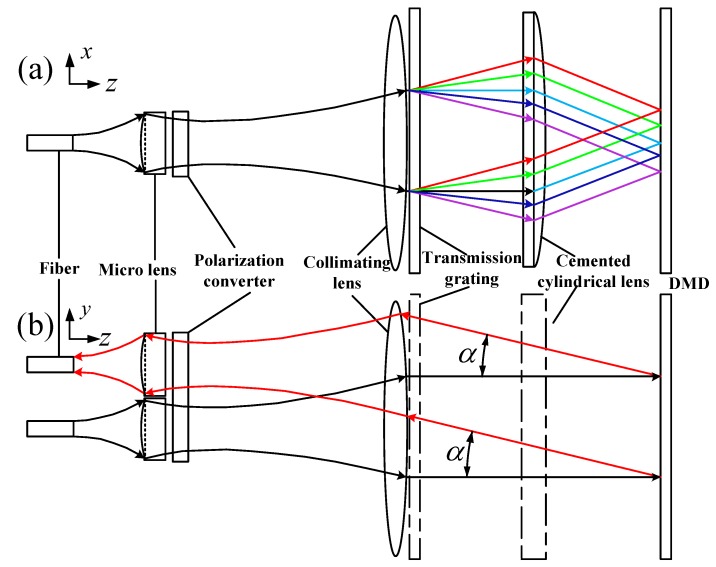
Layout of the filter optics: (**a**) The view in xz-plane showing light being de-multiplexed (**b**) The view in yz-plane showing light deflected by a DMD.

Figure 2a is the xz-plane view and Figure 2b is the yz-plane view. We simplify the optical configuration by ignoring the angle of a transmission grating, so that the optical axis shown in Figure 2 is a straight line. A microlens combining with a collimating lens converted an input divergent Gaussian beam into a 6 mm-diam parallel beam [19,20]. The collimated broad-band beam was angularly dispersed in the *x*-axis direction by a grating and then focused into an elliptical spot on a different area of the DMD after a cylindrical lens. The purpose of the elliptical spot was to obtain the minimum bandwidth and high diffraction efficiency [16]. The DMD was placed at the focal plane of both the cylindrical lens ($f_{2}$ = 140 mm) and the collimating lens ($f_{1}$ = 300 mm) to realize the function that each micromirror was controlled at the on or off states to select and steer arbitrary wavebands precisely to the output.

### 2.1. Diffraction Efficiency of DMD

**Figure 3:**
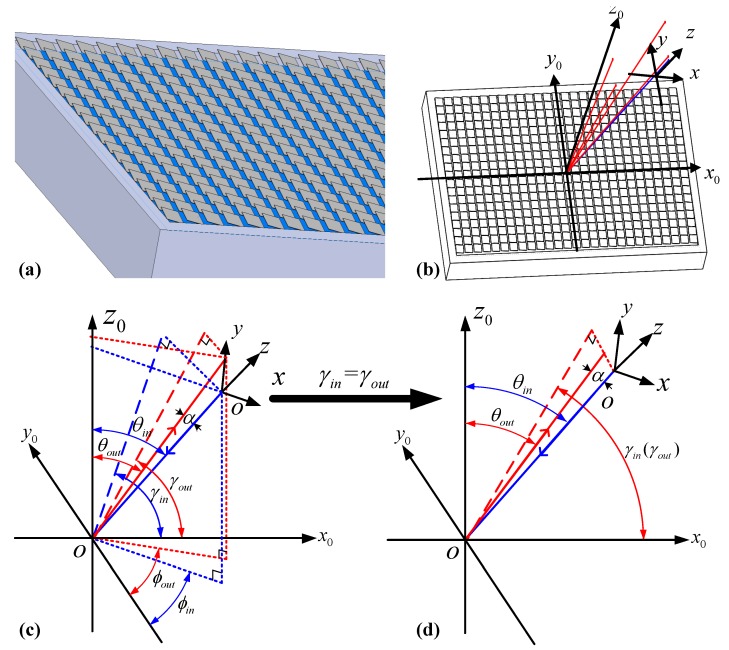
(**a**) Two-dimensional diffraction model of the DMD. (**b**) The incident beam and diffraction beam. (**c**) The coordinate system ($x_{0}$, $y_{0}$, $z_{0}$). (**d**) Distribution of the input and output beam when $\gamma_{in}=\gamma_{out}$.

In general, the long side of the DMD chip is aligned with the dispersion strip along the *x*-axis direction to maximize the spectrum utilization. The binary amplitude grating patterns are uploaded onto the DMD to control the corresponding micromirrors to tilt ±12° angle along their diagonals. The diffraction behavior of the several hundred thousand individually tilted micromirrors array as shown in Figure 3a is similar to a two-dimensional blazed grating. The diffraction distribution by the DMD and the coordinate system ($x_{0}$, $y_{0}$, $z_{0}$) is established in Figure 3b. The blue line represents an input beam and the red lines are the corresponding high-order diffraction beams in space. According to the 2D diffraction model [10], the diffraction angle of each order (p,q) is written as:

(1) $$\left\{\begin{array}{c} \phi_{out}\left(p,q\right)=\tan^{-1}\left(\frac{H\left(q\right)}{G\left(p\right)}\right)\\ \theta_{out}\left(p,q\right)=\sin^{-1}\left(\frac{G\left(p\right)}{\cos\phi_{out}\left(p,q\right)}\right), \end{array}\right.$$

where $H\left(q\right)=\frac{q\lambda }{\delta }+\sin\theta_{in}\cos\phi_{in}$, $G\left(p\right)=\frac{p\lambda }{\delta }+\sin\theta_{in}\cos\phi_{in}$. $\phi_{in}$ is the incident angle between the input plane and $y_{0}$-axis, $\theta_{in}$ is the angle between the incident beam and $z_{0}$-axis in output plane as shown in Figure 3c. $\phi_{out}$ and $\theta_{out}$ are defined in the same way. In Equation (1), (p,q)=0, ±1, ±2, ⋯ represent different diffraction orders. The diffraction distribution of an arbitrary order can be obtained when the incident angle $\phi_{in}$ and $\theta_{in}$ are provided.

As shown in Figure 3c, since the port distribution direction is perpendicular to the spectrum dispersion direction that is the $x_{0}$-axis direction, the incident angle needs to be adjusted to ensure the diffraction order with highest intensity is located in yoz-plane and routed back into an output port. The angle α is defined as that between input beam and diffraction beam. It is necessary to ensure $\gamma_{in}=\gamma_{out}$ in Figure 3d, with $\gamma_{in}$ and $\gamma_{out}$ being two angles between the projection of diffraction beam in $x_{0}oz_{0}$-plane and $x_{0}$-axis respectively. So the incident angles $\left(\theta_{in},\phi_{in}\right)$ satisfy the following condition:

(2) $$\frac{\cos\theta_{in}}{\sin\theta_{in}\sin\phi_{in}}=\frac{\cos\theta_{out}}{\sin\theta_{out}\sin\phi_{out}}.$$

**Figure 4:**
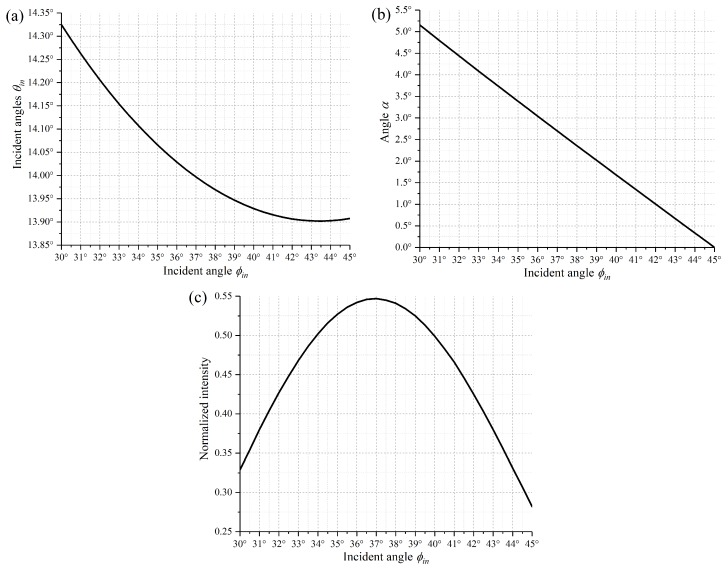
(**a**) Optimal incident angles $\left(\theta_{in}/\phi_{in}\right)$ for the optical system. (**b**) Dependence of the angle α on the incident angles $\theta_{in}$. (**c**) Dependence of the normalized intensity of output beam on incident angles $\theta_{in}$.

Based on the 2D diffraction model above, when a 1550 nm beam radiates on the +12° tilt DMD, it is noticed that the (−3,−3)-order diffraction beam always has a higher efficiency than the others. So multiple optimal incident angles according to Equations (1) and (2) are calculated and shown in Figure 4a. The angle α and normalized diffraction intensity of the (−3,−3)-order diffraction beam as a function of incident angles are presented in Figure 4b,c, respectively. Although the maximum normalized diffraction intensity can achieve 55%, the larger angle $\alpha =2.5^{\circ }$ worsens the optical aberration and insertion loss. So α is controlled at about 1° when $\theta_{in}$ = 13.91°, $\phi_{in}$ = 42°.

Table 1 gives the diffraction principal maximum beam when a 1550 nm beam radiating on the DMD at $\theta_{in}$ = 13.91°, $\phi_{in}$ = 42°, including the corresponding diffraction angle $\left(\theta_{out}/\phi_{out}\right)$ and relative intensity I(θ,ϕ) of each (p,q)-order. The brightest order of diffracted light is I(−3,−3) = 0.425, so that it is selected to couple into the output port while the other peaks are dramatically dropped out. The insertion loss by the DMD diffraction is around 3.7 dB.

### 2.2. Power Handling of Optical filter

**Figure 5:**
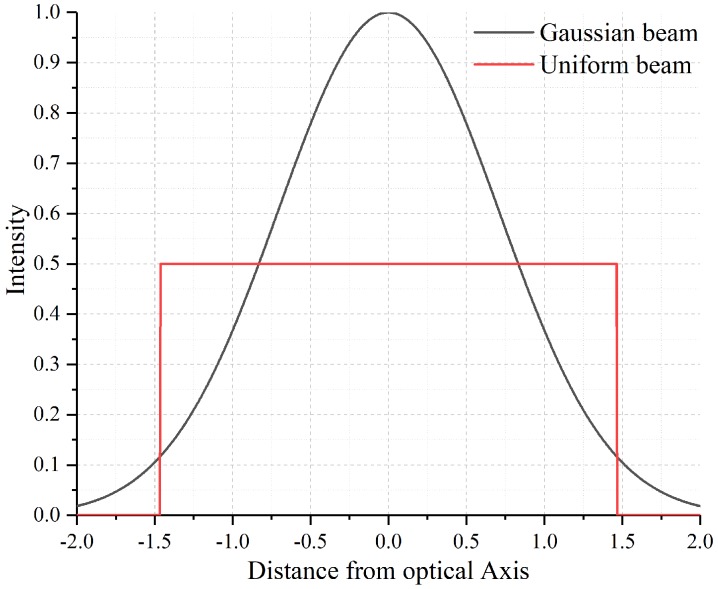
The beam intensity distribution of the Gaussian beam and uniform beam.

The power handling is one of the important specifications of optical filters. When the continuous wave (CW) laser illuminates a DMD, excessive energy absorption by on-surface aluminium-mirrors generally leads to the abnormal operation or even irreversible damage of the device. Therefore, it is necessary to keep the operating temperature below a critical point of 150 °C, and the average intensity cannot exceed 25 KW/cm^2^ in the visible band [18]. In general, the damage threshold depends on the illumination wavelength and intensity profile. For example, a damage threshold for 1550 nm is twice of 645 nm. As shown in Figure 5, a Gaussian beam has a maximum power density twice of the uniform beam when both beams have the same spot size and power. It is reported the damage threshold of the DMD for 1550 nm Gaussian beam is estimated to be about 25 KW/cm^2^ for CW-laser. Faustov [21] demonstrated that the measured threshold is up to 22 mW corresponding to 12 KW/cm^2^ when a He-Ne laser at 633 nm is focused onto a 13.7 μm × 13.7 μm-size micromirror. Furthermore, when an input laser at 1064 nm is below 30 mW ( 21 KW/cm^2^), micromirrors do not exhibit any visible damage. Schwarz et al. [22] also experimentally showed the damage threshold of a 19.3 KW/cm^2^ by 532 nm CW-laser.

The compressed light spot on the DMD means not only a narrower bandwidth for the filter, but also a higher energy density. The spot size on the DMD for this filter is measured to be 60 μm × 9 mm, so the max input power is about 135 W (50 dBm) corresponding to 25 KW/cm^2^. The power handling of commercialized WSSs (Waveshaper 16,000 A produced by Finisar Corporation) and LCoS based filters is 27 dBm input power in maximum. Therefore, DMD-based filters are an irreplaceable solution in high power situation.

## 3. Experimental Results and Discussion

**Figure 6:**
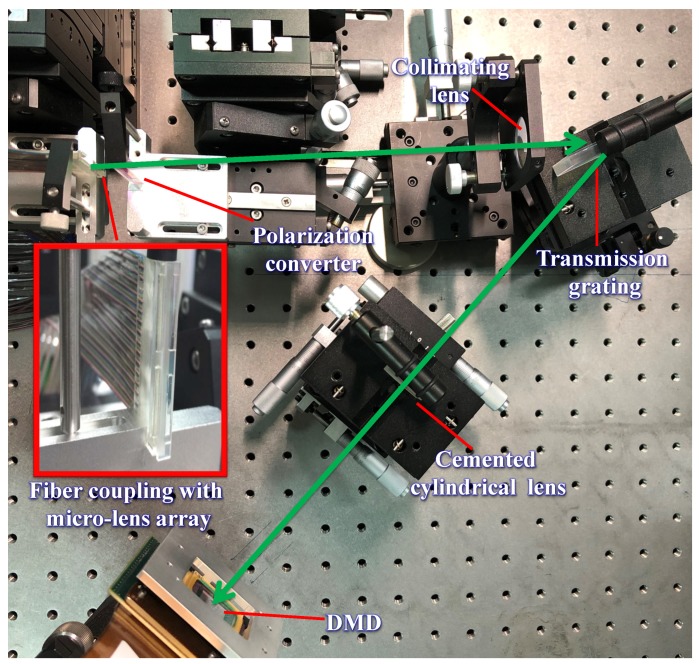
Arrangement of tunable optical filter.

**Figure 7:**
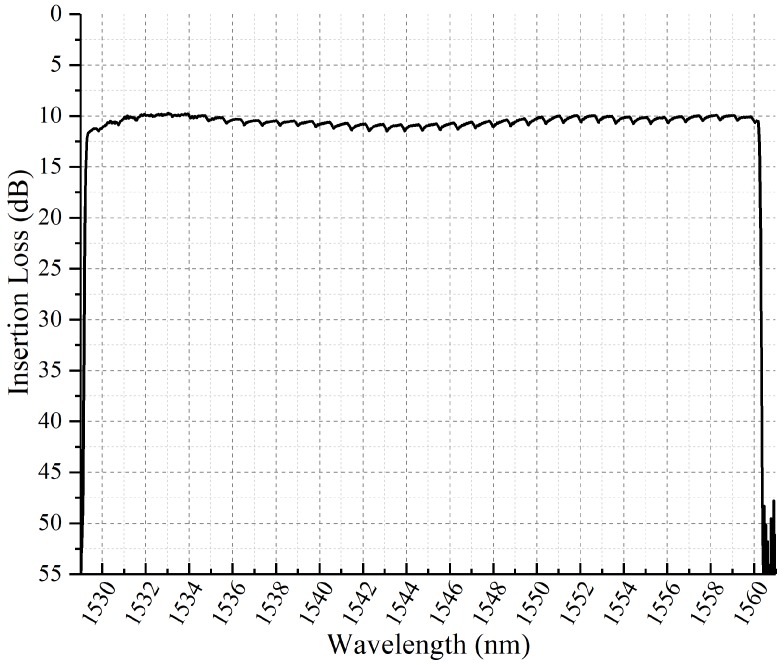
Total insertion loss as a function of C-band wavelength in filter system.

Figure 6 is the arrangement of the optical filter in experiment. The amplified spontaneous emission (ASE) light source in 1530–1560 nm was injected into the system as input signals. An optical spectrum measurement analyzer AQ6370C-YOKOGAWA was applied to measure the insertion loss, 3 dB-bandwidth and tuning resolution of the central wavelength. Figure 7 shows that the total loss was around 10 dB across the entire C-band with the ripples of 0.5 dB caused by the gap between micromirrors. The insertion loss mainly included 1.5 dB from the fiber-coupling microlens array, 0.5 dB from the polarization converter, 1.0 dB from the transmission grating and 4 dB from the DMD. In addition, when incident angle of the input beam at $\theta_{in}$ = 13.91°, $\phi_{in}$ = 42°, a mismatch between the focal plane of the cylindrical lens and the DMD caused 3 dB extra insertion loss. Although this 3 dB loss caused by oblique incident beam could be avoided by replacing the DMD with a 10.8 μm-pitch micromirror that had a 98% diffraction efficiency to vertical incident beam [10], it was not applied in the NIR-band without an anti-reflection coating, which would introduce more loss. The measured intrinsic polarization dependent loss (PDL) within the 12.5 GHz-bandwidth was less than 1 dB for the optical system. As the signal beam was diffracted by the transmission grating, a combination of conical diffraction and optical aberrations lead to the fluctuation of insertion loss of about 1 dB [16].

**Figure 8:**
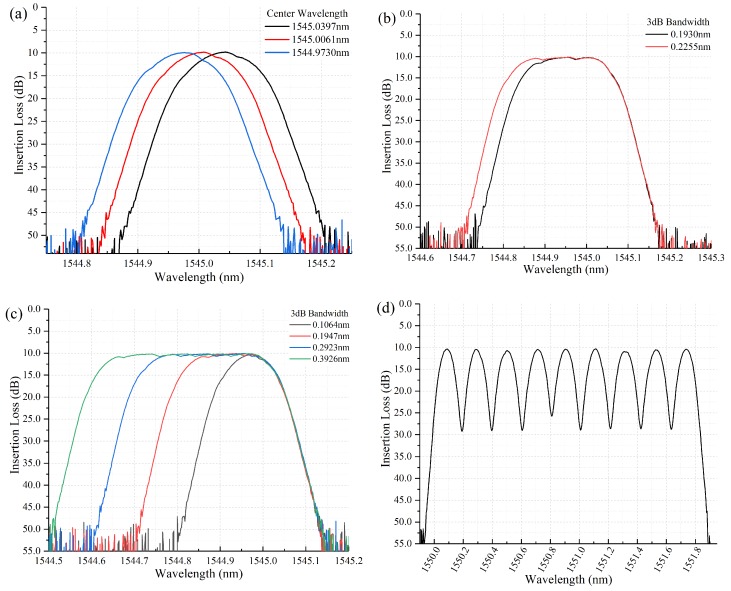
(**a**) The minimum tuning resolution of center wavelength in the filter. (**b**) The minimum tuning step of spectral bandwidth. (**c**) 3 dB-bandwidth from 12.5 GHz to 50 GHz with a step of 12.5 GHz. (**d**) Measured passband at 9 G.694.1 standard grid frequencies with 25 GHz channel separation.

Figure 8a shows that the center wavelength could be tuned flexibly in step of 0.033 nm with the 3 dB-bandwidth of 12.5 GHz. In Figure 8b, arbitrary wavebands could be filtered and configured at a minimum resolution of 0.033 nm. The 3 dB-passband could be adjusted flexibly from 12.5 GHz to 50 GHz by a step of 12.5 GHz in Figure 8c. The minimum filter bandwidth could achieve 12.5 GHz, however it was noticed that the top of wavelength profile was not flat enough. The measured passband at nine ITU-T G.694.1 standard grid frequencies with 25 GHz channel separation had about −15 ± 1 dB channel crosstalk in Table 2 and Figure 8d. Since the power falling edge of this filter was about 12 GHz spectral width in 20 dB, very narrowing spectral guard bands with small crosstalk could be set for 50 GHz and 100 GHz spaced ITU-T channels in the C-band.

**Figure 9:**
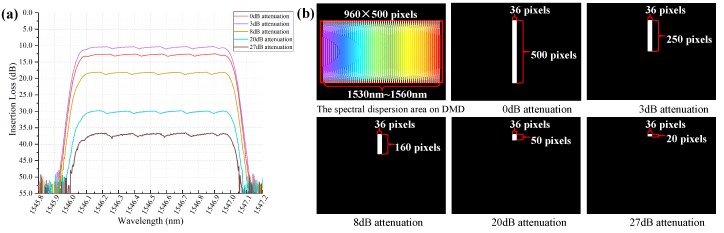
Optical power attenuation of the optical filter and the corresponding binary image.

In Figure 9a, the optical filter also provided a function of optical power attenuation. It was realized by controlling the corresponding micromirrors number in different locations to modulate the output luminous flux. The optical power attenuation could be adjusted from 0 dB to 40 dB flexibly with a resolution of 0.5 dB. Figure 9b shows the micromirrors information used to control the optical attenuation. This filter had 50 dBm maximum input power, and was an excellent equalizer for high power erbium-doped fiber amplifiers.

## 4. Conclusions

We propose and demonstrate a tunable optical filter with max 50 dBm input power, flexible central wavelength and bandwidth by employing a DMD processor into the system. The total insertion loss of this filter was about 10 dB across the entire C-band. The center wavelength and bandwidth of multi-channel could be tuned in the step of 0.033 nm independently. Although the minimum bandwidth could achieve 12.5 GHz, the performance of the channel crosstalk for 12.5 GHz and 25 GHz ITU grid especially still needs further improvement, as the spectral does not have an ideal flat-topped profile. In future work, we plan to optimize the minimum bandwidth by employing a specially designed cylindrical lens system to eliminate the spherical aberration and chromatic aberration, and further decrease the spot size in the *x*-axis on the DMD.

## Figures and Tables

**Table 1:** Irradiance maxima of light at 1550 nm radiating on the DMD over a large solid angle $\theta_{out}/\phi_{out}$, (I) of each diffraction order.

*p*/*q*	−4	−3	−2	−1
−4	22.8/45 (0.002)	18.6/30.4 (0.035)	16.1/9.9 (0.005)	-
−3	18.6/59.6 (0.019)	**13.2/45** **(0.425)**	9.7/16.5 (0.05)	10.0/−22.1 (0.009)
−2	16.1/80.1 (0.011)	9.7/73.5 (0.213)	3.872/45 (0.018)	4.7/−54 (0.004)
−1	−16.4/−76.6 (0.001)	−10.0/−67.9 (0.03)	−4.7/−36.1 (0.003)	-

**Table 2:** Channel crosstalk and offset level at nine grid frequencies ( 25 GHz channel separation).

Center Wavelength (nm)	1550.09	1550.30	1550.50	1550.71	1550.91	1551.11	1551.32	1551.53	1551.74
Offset Level (dB)	−0.557	−0.25	−0.067	−0.029	−0.502	−0.267	−0.124	0	−0.419
Channel Crosstalk (dB)	−16.345	−16.46	−16.132	−14.677	−14.223	−15.93	−15.739	−15.677	−16.319
